# Complete Genome Sequences of Serotype O Foot-and-Mouth Disease Viruses Recovered from Experimental Persistently Infected Cattle

**DOI:** 10.1128/genomeA.00606-15

**Published:** 2015-07-09

**Authors:** AravindhBabu R. Parthiban, Mana Mahapatra, Satya Parida

**Affiliations:** The Pirbright Institute, Pirbright, Woking, Surrey, United Kingdom

## Abstract

For the first time, the complete genome sequences of the six serotype O foot-and-mouth disease viruses from persistently infected carrier cattle are reported here. No consistent amino acid changes were found in these viruses obtained from persistently infected cattle compared with the complete genome of the parent virus that was used to infect the cattle.

## GENOME ANNOUNCEMENT

Foot-and-mouth disease (FMD) is a highly contagious acute viral disease of cloven-hoofed animals caused by a single-strand positive-sense RNA virus of the genus *Aphthovirus* in the *Picornaviridae* family. FMD virus (FMDV) can persist in the oropharynx of ruminants for years following the resolution of acute infection (1). As live virus can be isolated from the oropharyngeal fluids of such persistently infected animals, the potential escape of viruses from these animals to initiate a new outbreak cannot be ruled out. The mechanism and factors responsible for the persistence of FMDV are yet unclear. The availability of the complete genome sequences of the virus from persistently infected animals may help better understand the viral determinants, if any, that are responsible for causing cattle persistently infected with FMDV.

Four FMDV vaccine challenge experiments were previously conducted in the animal isolation unit (biosafety level 3 [BSL3] containment) at The Pirbright Institute to study FMDV infection in vaccinated animals (2–6). The persistently infected cattle were selected by isolating viruses and detecting genomes from the oropharyngeal fluid at 28 days postchallenge and kept up to 168 days postchallenge (5). The complete genome sequences of FMD viruses were carried out directly from RNA obtained from probang fluids of three persistently infected cattle at six different time points that were experimentally infected with O/UKG/35/2001 FMDV. The complete genome sequences of these viruses were compared with those of the parent virus that had been used for infecting the cattle. Viral RNA was sequenced by generating overlapping amplicons, which were gel purified and sequenced using an ABI-3730 automated sequencer (Applied Biosystems), and the contigs were assembled using SeqMan version 7.0 (DNAStar Lasergene, USA) (7).

The complete genomes of the challenge virus and the persistent viruses are 8,183 nucleotides in length. The genomes contain a 1,091-nucleotide-long untranslated region (UTR) at the 5′ end, followed by a 6,999-nucleotide-long polyprotein-coding region and a 94-nucleotide-long UTR at the 3′ end, excluding the poly(A) tail. The poly(C) within the 5′ UTR and the poly(A) tail at the 3′ end of FMDV are highly varied; 10 nucleotides of C were substituted during sequence analysis and no As were included. The nucleotide identity between the challenge and carrier viruses ranged between 98.90% and 99.77%, and the amino acid identity ranged between 99.18% and 99.96%. There were no consistent changes found in the amino acid sequences between the challenge virus and the viruses from persistently infected cattle. This observation indicates that there is no viral determinant that plays a role in persistently infecting cattle.

### Nucleotide sequence accession numbers. 

The complete genomes of the FMD viruses recovered from persistently infected cattle and the virus used to challenge cattle have been deposited in GenBank under the accession numbers KR265072, KR265073, KR265074, JX947859, KR401161, JX947858, and KR265075.
